# Supplementary material for: Spatially confined lignin nanospheres for biocatalytic ester synthesis in aqueous media
Source: Nat Commun. 2018 Jun 12;9:2300. doi: 10.1038/s41467-018-04715-6 (PMC5997711; doi:10.1038/s41467-018-04715-6)
Supplement: Supplementary file 1 — Supplementary Information [file 41467_2018_4715_MOESM1_ESM.pdf]

Supplementary Information

Spatially Confined Lignin Nanospheres for Biocatalytic Ester Synthesis in Aqueous Media

Sipponen et al.

This PDF file includes:

Supplementary Notes 1–6

Supplementary Figures 1–12

Supplementary Tables 1–5

Supplementary References

Contents

Supplementary Note 1. Lignin characterization 1

Supplementary Note 2. Enzyme characterization and immobilization 4

Supplementary Note 3. Electron microscopy of CLP dispersions 8

Supplementary Note 4. QCM-D study of multilayer adsorption 9

Supplementary Note 5. Aqueous esterification catalyzed by immobilized hydrolases 11

Supplementary Note 6. Statistical analysis 17

Supplementary References 18

Supplementary Note 1. Lignin characterization

Softwood kraft lignin (SKL) served as a raw material for the preparation of colloidal lignin particles (CLPs) and cationic lignin nanospheres. To provide sufficient structural view of this polydisperse methoxylated polyphenol, we subjected SKL to a series of characterizations. The functional groups from the ^{13}C NMR and ^{31}P NMR analyses show the predominance of hydroxyl groups, with phenolic hydroxyl groups being the most abundant (Supplementary Table 1). The occurrence of β–*O*-4 bonds was only five per 100 Ar, while in native softwood lignin their occurrence is 50% or more of all interunit linkages.[1] Methoxyl content of SKL indicates 25% demethylation during kraft pulping. Overall, these values, as well as the weight average molecular weight (M_w=5250 g mol^{-1}, M_w/M_n=4.4) and molar mass distribution (Supplementary Figure 1), agree well with published data for softwood kraft lignin.[2,3]

Supplementary Table 1 | Analysis of various moieties of SKL and corresponding cationic lignin by quantitative ^{13}C and ^{31}P NMR spectroscopy experiments.

Moiety	^{31}P	^{13}C	
	mmol g^{-1}	mmol g^{-1}	Per 100Ar
Total OH	6.32	6.44	107.0
Aliphatic OH	1.89	2.61	52.3
Total phenolic OH	4.05	3.56	59.2
5-free guaiacyl OH	1.94	1.70	28.3
5-substituted guaiacyl OH	1.80	1.87	31.0
p-hydroxyphenyl OH	0.31	nd	nd
OMe	-	4.37	75.3
Pinoresinol	-	0.10	1.73
Phenylcoumaran	-	0.12	2.06
β-O-4	-	0.24	4.67
β-5	-	0.12	2.06
β-β	-	0.05	0.87
G	-	2.46	41.0
H	-	0.13	2.31
Non-conjugated COOR	-	0.69	12.2
Conjugated COOR	-	0.05	0.87
Conjugated CO	-	0.22	3.83
Non-conjugated CO	-	0.37	6.31
Sugars	-	0.05	0.80

Reported values are mean values of two replicates with percentage errors generally <5% relative to the mean.

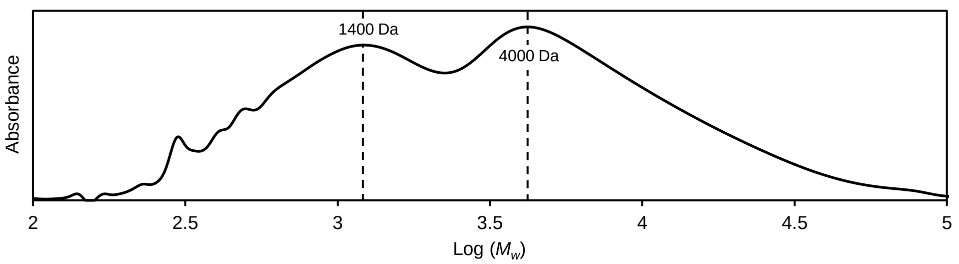

Supplementary Figure 1 | Gel permeation chromatography trace of acetylated SKL in THF.

Despite its heterogeneous, chemically altered structure, SKL was converted to spherical CLPs in 80% mass recovery yield. Additionally, cationic lignin was synthesized in a homogeneous reaction in which SKL was reacted with glycidyl trimethyl ammonium chloride (GTMAC) under alkaline conditions. The reaction products were purified by dialysis against deionized water, and the pH 7-soluble fraction was recovered for further analysis. Based on the ^{31}P NMR data for phenolic hydroxyls (Supplementary Table 2) the cationization reaction converted 65% of phenolic hydroxyl groups of SKL to corresponding hydroxypropyls, each carrying a positively charged quaternary amine group. The estimated quantity of cationic groups (2.64 mmol/g) exceeded the total amount of carboxylic and phenolic hydroxyls (2.03 mmol/g).

Supplementary Table 2 | ^{31}P NMR analysis of lignin. Amounts for SKL and the corresponding cationized lignin (Catlig) are in mmol g^{-1}.

Lignin	Aliphatic	COOH	H	G	Total phenolic
SKL^b	1.89±0.05	0.57±0.07	0.31±0.02	3.74±0.02	4.05±0.04
Catlig^b	2.64±0.04	0.61±0.23	0.10±0.02	1.32±0.05	1.42±0.03

Pooled percentage errors relative to the means (N=2) were 1% (pine kraft lignin) and 4% (Catlig).

Alkylated lignins were characterized by infrared spectroscopy in comparison to the spectra of unmodified SKL and Catlig (Supplementary Figure 2). While absent in SKL and Catlig, the predominant C=O stretch of acetyl and butyric groups were observed from bands at 1736 and 1760 cm^{-1}. Acetyl groups could also be traced from the band at 1368 cm^{-1} that is assigned to aliphatic C–H stretch in CH_3 (not methoxyl). Catlig exhibited elevated band intensities for

aliphatic C–H deformations at 1470 cm^{-1} and aromatic ring vibrations at 1510 and 1270 cm^{-1}, while for Catlig the band at 1032 cm^{-1} (aromatic C–H in-plane deformation) was distinctly stronger when compared to those of other lignins. Overall, these alterations result from etherification of the aliphatic and phenolic hydroxyl groups, as also seen from NMR analyses.

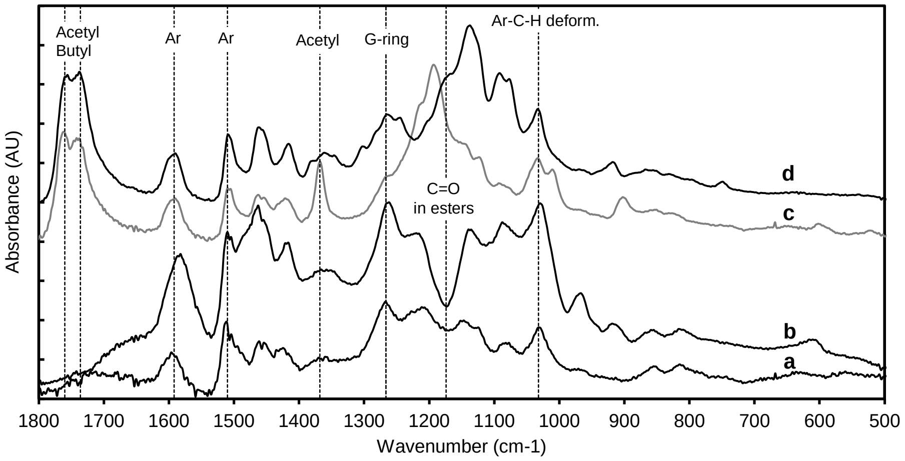

Supplementary Figure 2 | Fingerprint region of the ATR-FTIR spectra of lignins. **a**, SKL **b**, Catlig **c**, acetylated SKL **d**, butyrated SKL. The bands were assigned based on the prior literature.[4] SKL, pine kraft lignin. Catlig, cationic lignin.

A heteronuclear single quantum coherence spectroscopy (HSQC) ^{1}H–^{13}C spectrum of acetylated SKL showed the expected absence of C2–H and C6–H syringyl signals, while a contours were observed for aromatic C–H correlation signals in guaiacyl units (Supplementary Figure 3). Distinct signals for methoxyl, acetyl, and Cγ-H moieties were present, but only traces of β-*O*-4 and β-β were observed, which accords with the quantitative ^{13}C NMR results discussed above.

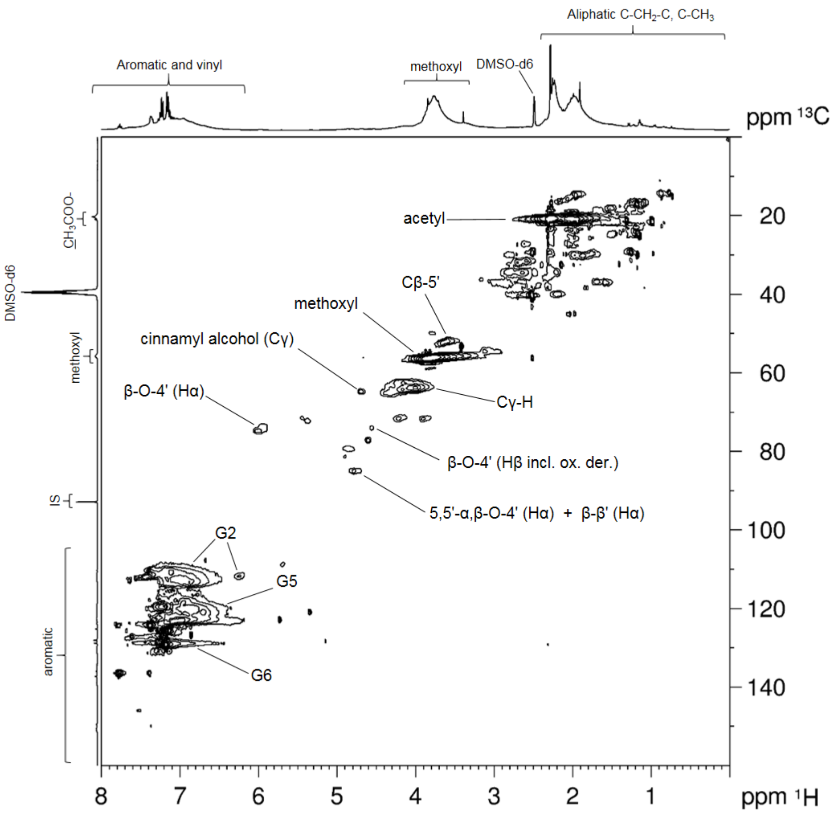

Supplementary Figure 3 | A heteronuclear single-quantum coherence (HSQC) spectrum of acetylated SKL recorded in DMSO-d6. Contours were assigned according to prior literature.[5,6] Internal standard (IS, trioxane) was not used in the HSQC experiment. SKL, softwood kraft lignin.

Supplementary Note 2. Enzyme characterization and immobilization

Lipase M from *Mucor javanicus*, *Humicola insolens* cutinase (HiC) (Novozymes, China), and *Candida antarctica* lipase B (CALB, immobilized on acrylic-resin) were assayed for hydrolytic activity using aqueous mixture of tributyrin as substrate. The results are given in Supplementary Table 3. The hydrolytic activities were in the order HiC << acrylic-resin immobilized CALB < Lipase M. These values served as a guideline when deciding the amounts of catalysts for comparative esterification experiments in aqueous-organic biphasic. SDS-PAGE analysis of Lipase M showed a distinct band at around 33 kDa (Supplementary Figure 4), suggesting significantly larger size than the previously estimated 21 kDa.[7] The size of CALB is also 33 kDa, while HiC exhibited a band at 23 kDa, the smaller molecular weight

being typical to microbial cutinases.[8] HiC was received in liquid media and was purified via three ultrafiltration steps using a Vivaspin 20 PES twin membrane ultrafiltration unit (Sartorius, Germany) with a *MWCO* of 10 kDa before further use. The analysis of the purified enzyme *via* SDS-PAGE showed a 95% purity. After purification, the protein concentration was 12.1 g l^{-1}, determined *via* the BSA assay (BioRad protein assay, Bio-Rad Laboratories GmbH, Vienna, Austria).

Supplementary Table 3 | Hydrolytic activities (in nkat mg^{-1} enzyme preparation, dry basis) of the enzymes used in the current work. Assay conditions: 30 min vigorously stirred reaction at 40 °C in tributyrin/aqueous 0.2 M Tris–HCl buffer (pH 7.0) mixture. More details are given in Methods.

Enzyme preparation	Amount of enzyme in the assay (mg)[a]	Activity (nkat mg^{-1})
Lipase M	15.6	16.3 ± 0.6
HiC	0.48	69500 ± 6400
CALB-acrylic resin	15.3	106.4 ± 8.4

[a]: The amount of enzyme preparation in 1 ml aliquot added to the reaction mixture.

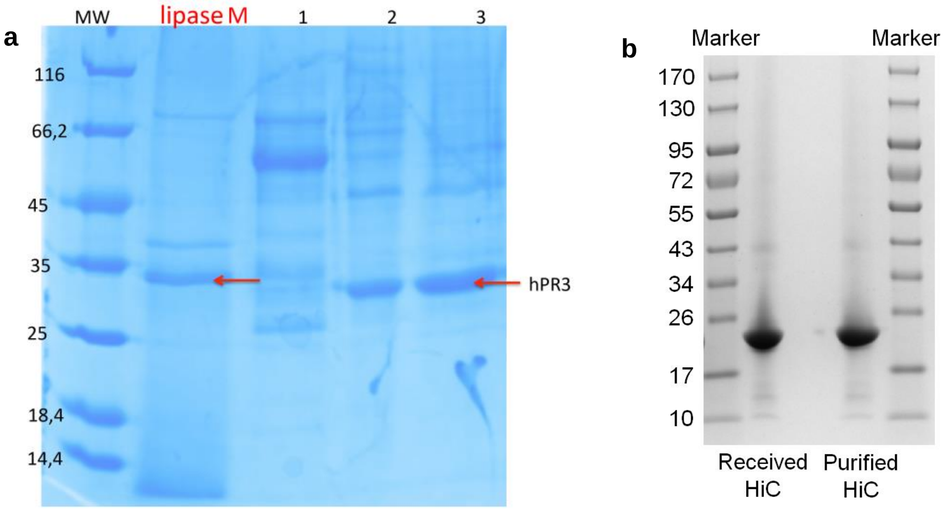

Supplementary Figure 4 | SDS-PAGE analysis of hydrolases. a, Lipase M compared to human PR3 protein (M_w of 26 kDa + 2 kDa for n-glycosylation) at various purification degrees in lanes 1–3. **b**, HiC before and after purification.

The efficiency of enzyme immobilization was assessed from protein mass balance (Bradford method, BSA standards) determined for the spatial confinement approach and the mere

calcium alginate entrapment of Lipase M separately. During the first step, 91% of Lipase M adsorbed on c-CLPs, while after the second step 96% of the initial amount of enzyme was entrapped in the hydrogel matrix (Supplementary Figure 5). The entrapment efficiency of Lipase M in the absence of c-CLPs was essentially similar 97%. These results support earlier observations indicating that enzyme immobilization in calcium alginate occurs with nearly complete protein entrapment.[9]

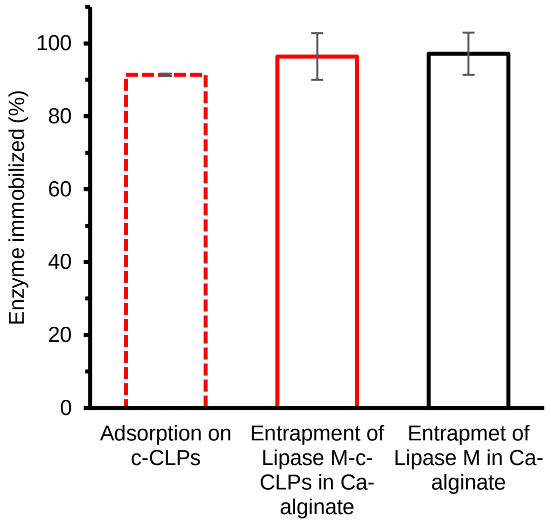

Supplementary Figure 5. | Efficiency of Lipase M immobilization as determined from the protein mass balance. In the spatial confinement approach, soluble protein was determined after adsorption onto c-CLPs, and after entrapment in calcium alginate. The percentage of Lipase M entrapped in calcium alginate without cationic lignin nanospheres is shown for comparison. Error bars represent two standard deviations from the mean values (two experimental replicates).

Materials used in the fabrication of immobilized biocatalysts by entrapping Lipase M in calcium alginate hydrogel with various surface-active materials are shown in Supplementary Table 4. Moisture content of the biocatalyst beads and the amounts used in the esterification experiments are indicated. The stability of spatially confined Lipase M was assessed by stirring the dried biocatalyst beads in aqueous buffer solutions for the duration of 72 h. The appearance of the mixtures are shown in Supplementary Figure 6.

Supplementary Table 4 | Fabrication of immobilized biocatalysts by adsorption of Lipase M or HiC on surface-active materials and subsequent entrapment into calcium alginate hydrogel matrix. The alginate beads formed were dried overnight and the residual moisture content was analyzed gravimetrically from separate samples dried at 105 ºC to constant weight. Weight fractions of enzyme and surface-active materials are indicated on dry weight basis.

	Surface active material	Enzyme proportion (wt% d.b.)[a]	Surface-act. material (wt%)	Dry content (wt%)	Amount used in esterification (mg, f.w.)		hexane : H_2O v/v
					Replicate 1	Replicate 2	
Lipase M	None	5.3	-	80.4	514.2	507.7	1:1
	SKL	5.3	5.5	83.5	510.3	511.4	1:1
	Catlig+SKL	5.2	0.31+5.3	83.5	508.0	512.8	1:1
	CLPs	5.4	3.5	83.5	508.2	512.0	1:1
	c-CLPs (batch 1)	5.4	0.32+3.4[b]	72.5	509.0	514.1	1:1
	c-CLPs (batch 2)	5.4	0.30+3.4[b]	84.5	514.2	508.6	1:1
	c-CLPs (batch 3)	5.4	0.30+3.4[b]	88.4	508.9	515.1	1:1
	Bu-CLPs	5.4	3.6	78.4	509.3	516.6	1:1
	Bu-CLPs	8.3	8.4	83.5	511.9	509.3	1:1
	c-Bu-CLPs	5.4	0.30+3.5[b]	78.7	513.3	513.0	1:1
	c-Bu-CLPs	5.4	0.48+8.3	81.6	512.8	507.4	1:1
	Catlig	5.3	0.61	83.6	512.4	510.6	1:1
	Tween 80	5.3	0.61	82.7	515.6	509.6	1:1
	SDS	5.3	0.61	82.2	506.8	513.6	1:1
	DDMA	5.3	0.61	83.8	507.9	515.0	1:1
	c-CLPs (batch 2)	5.4	0.30+3.4[b]	84.5	2010.7	2009.9	1:9
	c-CLPs (batch 2)				1529.5	1525.9	1:1
	c-CLPs (batch 1)	5.4	0.32+3.4[b]	72.5	506.3±4.7 (kinetics)		1:1
	Catlig+CLP+CWP	5.1	0.25+2.0+1.0	83.2	513.2	509.5	1:1
HiC					26.3	26.1	1:1
	c-CLPs	2.84	0.28+3.5[b]	76.4	103.6	103.2	1:9
					104.0	104.9	1:1
					104.0	104.3	1:1[c]
	Tween 80	2.94	0.32	78.0	25.5	26.2	1:1
CALB	Acrylic resin	n.a.	n.a.	n.a.	5.03	4.99	1:1
		n.a.	n.a.	n.a.	20.3	20.5	1:9

[a]:Theoretical amount. The protein content of Lipase M was 48±3% (Bradford assay, BSA standards). [b]:weight proportions for Catlig+CLPs. [c]: Reaction carried out after 251 days storage of the biocatalysts at 4 °C. d.b., dry basis. f.w., fresh weight. n.a., not analyzed. SKL, pine kraft lignin. Catlig, cationic lignin. CLP, colloidal lignin particle. CWP, Carnauba wax particle. Bu-, butyrated. SDS, sodiumdodecyl sulfate, DDMA, didodecyldimethylammonium bromide. HiC, *Humicola insolens* cutinase.

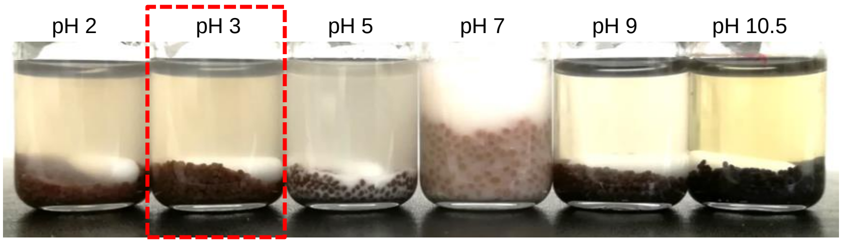

Supplementary Figure 6 | pH-stability of biocatalysts. Appearance of alginate beads containing spatially immobilized Lipase M after 72 h magnetic stirring at 21 °C in 0.2 M aqueous buffers solutions (pH 2 HCl–KCl, pH 3 HCl–glycine (pH used in the esterification reactions), pH 5 and pH 7 sodium phosphate, pH 9 and pH 10.5 sodium borate).

Supplementary Note 3. Electron microscopy of CLP dispersions

CLPs tend to agglomerate on surfaces upon drying from water. This was observed from TEM images in Supplementary Figure 7a,b,d that show clustered particles irrespective of their origin (regular CLPs or Bu-CLPs). Volume shrinking due to the drying of the alginate beads caused steric immobilization of the assembled particles (Supplementary Figure 7c). When cationic lignin nanospheres were used to anchor the enzymes prior to the entrapment, larger 1–2 µm particle clusters were observed (Figure 3c, k–m of the main article and Supplementary Figure 7e–f). It is assumed that these clusters are partially preformed in the sodium alginate mixture before the formation of the hydrogel by calcium induced cross-linking, while the confined clusters are further reinforced by the removal of free water. With Lipase M adsorbed on c-Bu-CLPs, several distinct clusters comprising of approximately 10 individual particles were observed (Supplementary Figure 7e–f). The cross-sections exposing interiors of Bu-CLPs show that these are compact particles without observable hollow core-shell structure.

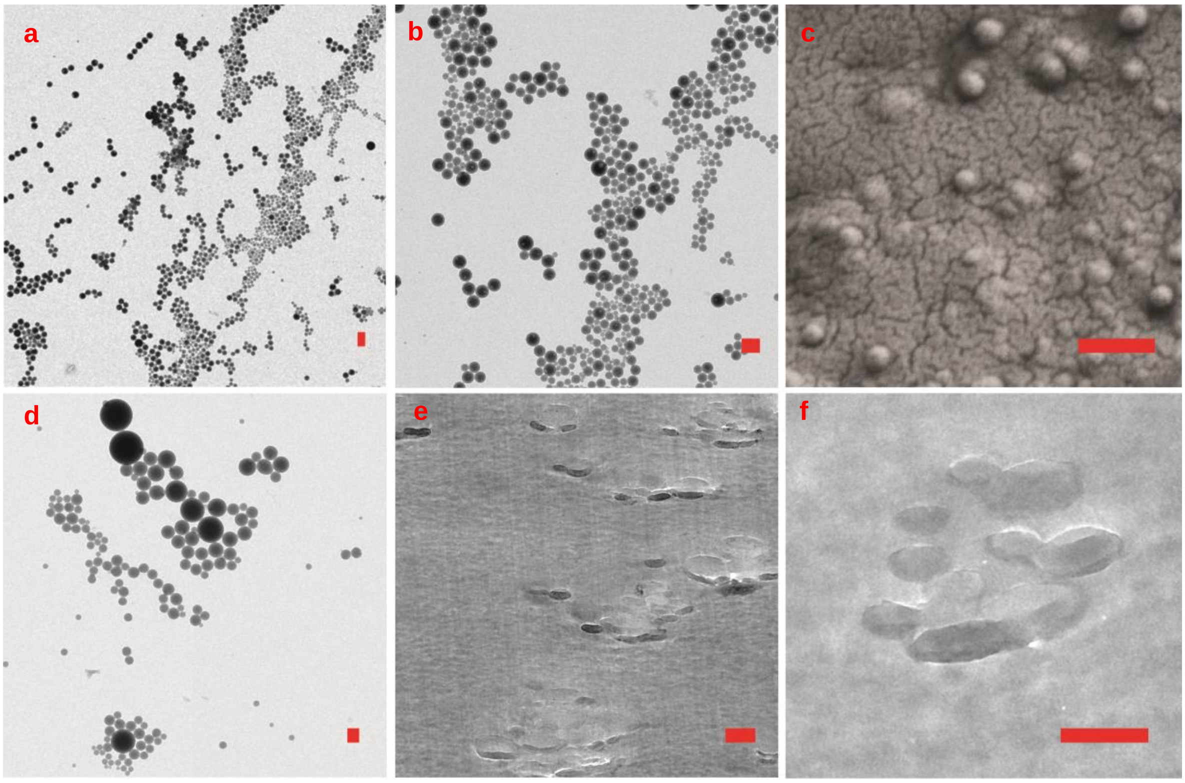

Supplementary Figure 7 | Electron microscopy images of colloidal lignin particles as such and after entrapment in calcium alginate. TEM images showing aggregation of CLPs (**a-b**) and Bu-CLPs (**d**) upon drying from aqueous dispersion. **c, e, f,** Particles associated with Lipase M inside calcium alginate beads. **c**, SEM image of exposed cross section of an alginate bead containing Lipase M adsorbed on CLPs. **e-f**, TEM images of thin films cut using a microtome from alginate beads containing Lipase M adsorbed on c-Bu-CLPs. Scale bars = 200 nm.

Supplementary Note 4. QCM-D study of multilayer adsorption

Layer-by-layer (LbL) assembly was tested as a possible approach to fabricate multilayer films containing lipase layers embedded between cationic lignin nanosphere layers. To determine affinity of Lipase M to adsorb on cationic lignin surfaces, cellulose nanofibril (CNF) thin films were deposited on QCM-D gold sensors.[10] An E-4 QCM-D instrument (QSense AB, Västra Frölunda, Sweden) was used at a flow rate of 0.1 ml min^{-1} to follow adsorption of 50 mg l^{-1} aqueous lignin or enzyme solutions or dispersions at pH 6 on CNF thin films. As detected by QCM-D, adsorption of Catlig on CNF surfaces proceeded slowly (Supplementary Figure 8a). After reaching a plateau, the crystals were rinsed with deionized water, and CLPs and Carnauba wax particles (CWPs) dispersions were pumped on the QCM-D cells. A rapid mass

increment occurred due to the electrostatic adsorption of CLPs on Catlig surfaces. While adsorption of CWPs was initially only slightly slower, it continued after the initial fast step and with a steeper slope than observed for CLPs. It appears that in addition to electrostatically driven adsorption, the rate-limiting step is the entrapment of colloidal particles in the porous CNF matrix. With adsorption of Lipase M, entrapment may have occurred into the porous nanofiber matrix, since a significant extent of adsorption occurred also on CNF surface without Catlig (Supplementary Figure 8b). When Lipase M was adsorbed after prior coating of the CNF film with Catlig, the adsorption rate for the enzyme was slower. However, there was a rapid initial adsorption stage indicating electrostatic interactions between Lipase M and surface-deposited cationic lignin. CLPs and CWPs adsorbed on CNF surfaces were imaged using atomic force microscopy (AFM) in tapping mode. Compared to the CNF surface without added particles, CLPs and CWPs were clearly observed in the AFM images (Supplementary Figure 8c–e). There was a higher number of CWPs than CLPs on the surfaces, but the latter were larger and less uniformly distributed.

For the fabrication of immobilized biocatalyst *via* LbL assembly, microcrystalline cellulose (MCC) microspheres (JRS Pharma Oy, Finland) with hydrodynamic diameter of 203±2 μm (volume weighted mean, N=6) were used as templates. The adsorption sequence consisted of three multilayers of [Cationic lignin/Lipase M/Cationic lignin/Bu-CLPs] followed by a single layer of sodium alginate, and final immersion in aqueous calcium chloride. All solutions or dispersions contained 1 g l^{-1} dry material. The LbL-coated MCC spheres were washed after each 15 min adsorption step by soaking in deionized water, and finally dried under ambient conditions at 21 ºC.

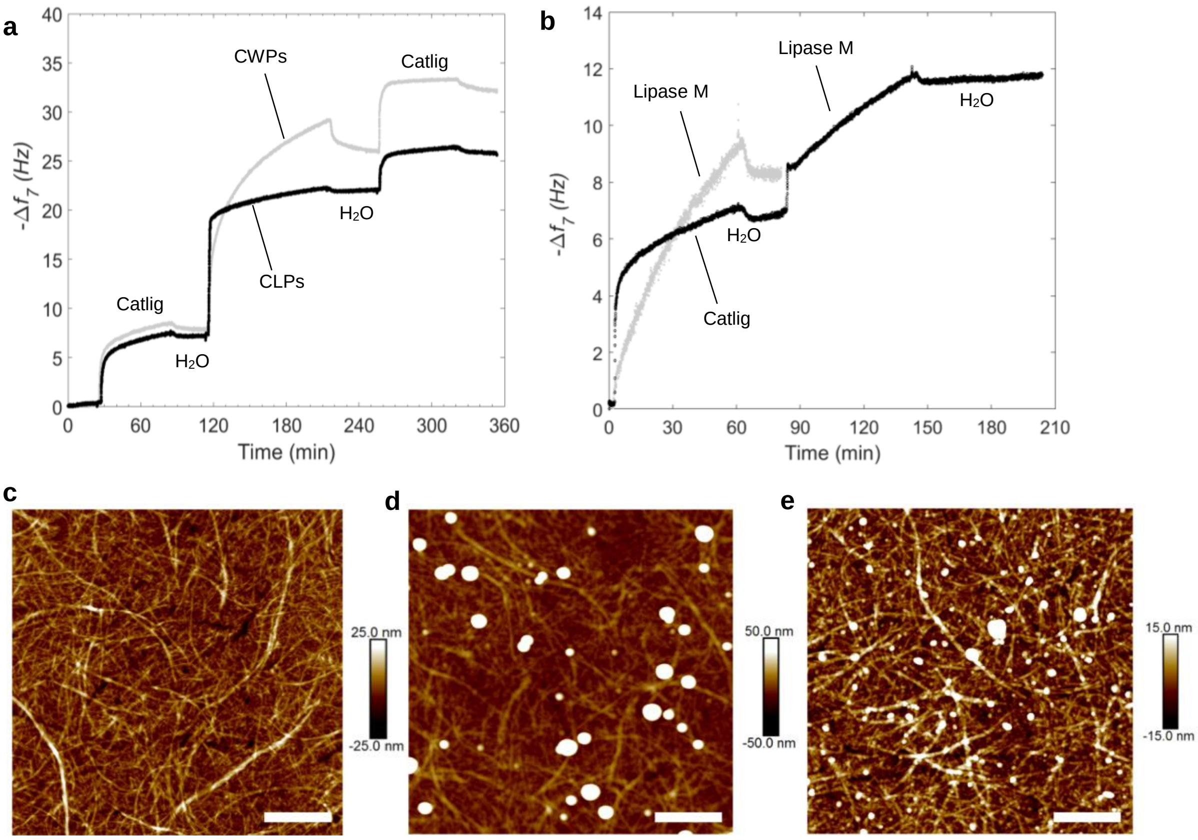

Supplementary Figure 8 | QCM-D study of layer-by-layer adsorption. a, Adsorption of CLPs and CWPs on cellulose nanofibril thin film deposited on gold-coated QCM-D sensor. **b**, Adsorption of Lipase M from *Mucor javanicus* on cationic lignin surfaces on CNF thin films. QCM-D curves show changes in the seventh overtone frequency due to the adsorption. AFM height images of **c**, CNF thin film as such and after adsorption of **d**, Catlig and CLPs; **e**, Carnauba CWPs. Scale bars = 1 µm. CLP, colloidal lignin particle; CWP, Carnauba wax particle; Catlig, cationic lignin. AFM, atomic force microscopy.

Supplementary Note 5. Aqueous esterification catalyzed by immobilized hydrolases

Aqueous esterification of butyl butyrate was carried out initially in the absence of any organic phase. The reaction mixture in a 10 ml glass vial contained 500 mg of the immobilized biocatalyst (Lipase M in LbL-coated MCC spheres or entrapped in calcium alginate) and 2.5 ml of aqueous solution of butanol and butyric acid at pH 3. The concentrations of n-butanol and n-butyric acid were 0.15 M and 0.05 M according to the optimised molar ratio constant.[11] The capped vial was subjected to 125 rpm orbital shaking at 40 °C. The products were extracted after 96 h reaction with 2 ml of n-hexane at 0 °C (60 min, 125 rpm shaking).

Butyl butyrate was detected in trace concentration after 96 h reaction compared to notably higher levels from the biphasic aqueous-organic esterification reaction (Supplementary Figure 9a–b). This result nonetheless indicates that it is possible to drive the reaction towards esterification in the absence of organic solvents with spatially confined lipases. Though promising result as such, leaching of cationic lignin occurred during the aqueous reaction, impeding the repeated use of the biocatalyst. This prompted shifting the focus on immobilization of lipases by entrapment in hydrogel matrices.

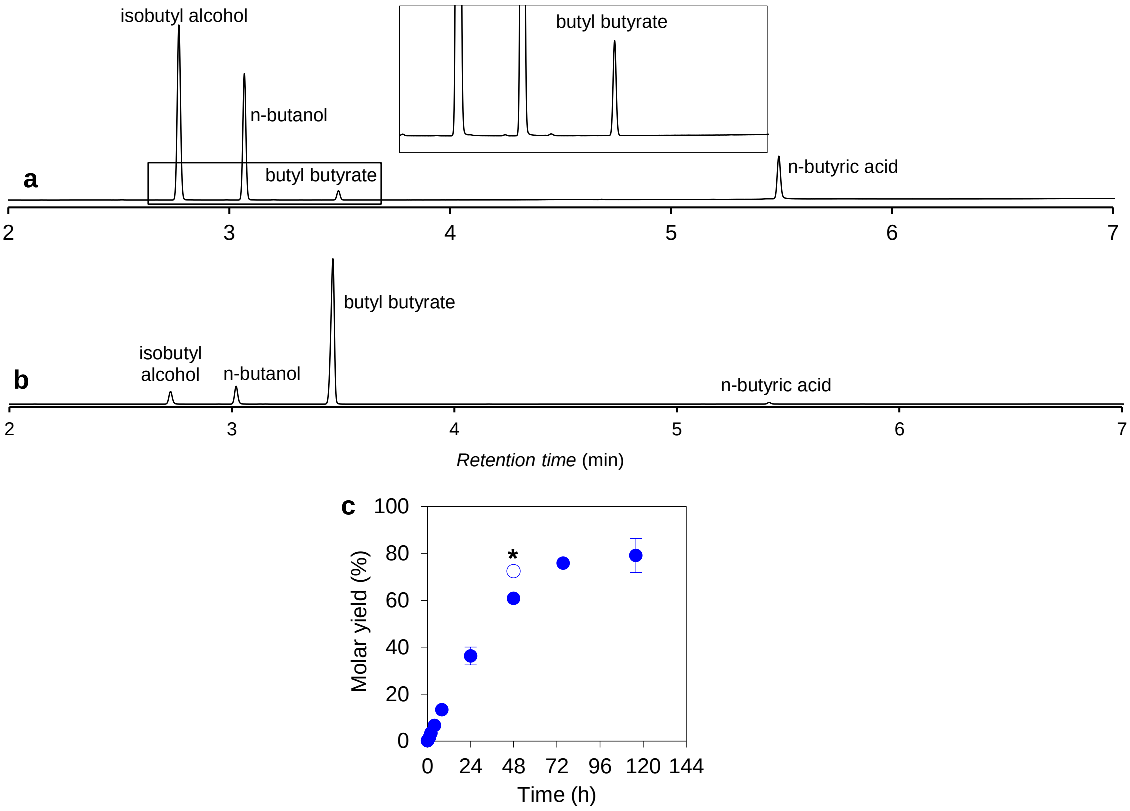

Supplementary Figure 9 | Gas chromatography-flame ionization detection of butyl butyrate from enzymatic esterification reactions. a, Solvent-free aqueous esterification reaction catalyzed by LbL-coated MCC spheres. The LbL-coating contained approximately 2% of Lipase M relative to the dry weight of the MCC spheres. **b**, Biphasic reaction (hexane:water 1:9 v/v) catalyzed by spatially confined biocatalyst containing Lipase M and cationic lignin nanospheres. **c**, Kinetics of the biphasic reaction (hexane:water 1:1 v/v) catalyzed by spatially the confined biocatalyst, with the open circle marked with an asterisk denoting experiments carried out with a threefold enzyme to substrate ratio compared to the other data points. Reactions at 40 ºC (pH 3) were carried out during 24 h continuous agitation at 125 rpm (**a**) or 200 rpm (**b–c**). The organic phase analyzed in **b** was diluted by a factor of two before adding isobutyl alcohol and as internal standard in GC-FID analysis. Error bars represent two standard deviations from the mean values (two replicates).

Effect of varied weight fractions of hydrolases and surface-active materials on esterification activity was assessed in immobilization of Lipase M. At constant weight fraction of Lipase M in the alginate beads, replacement of c-CLPs (series F in Supplementary Figure 10a) by c-Bu-CLPs (series C in Supplementary Figure 10a) caused a reduction in the butyl butyrate yield from 35% to 28% in the first 24 h reaction. While maintaining the proportion of Lipase M constant, increasing the proportion of c-Bu-CLPs from 3.5% (series C in Supplementary Figure 10a) to 8.3% (series D in Supplementary Figure 10a) did not increase the detected synthetic activity. For anionic Bu-CLPs, a 56% increase in the weight fraction of Lipase M from 5.4% (series A in Supplementary Figure 10a) to 8.4% (series B in Supplementary Figure 10a) caused only a slight increase in butyl butyrate yield from 25% to 33%. An attempt to increase hydrophobicity of the alginate beads by replacing one third of the amount of CLPs used in the spatially confined biocatalyst (series F Supplementary Figure 10a) by CWPs (series E in Supplementary Figure 10a) reduced synthetic activity to a level that was comparable to that exhibited by the immobilization system with 8.3% weight fraction of c-Bu-CLPs. Overall, these results indicate that it is preferable to use smaller colloidal particles that occupy a lower volume, form confined reaction clusters in the entrapped hydrogel, and provide larger surface area for the adsorbed hydrolases.

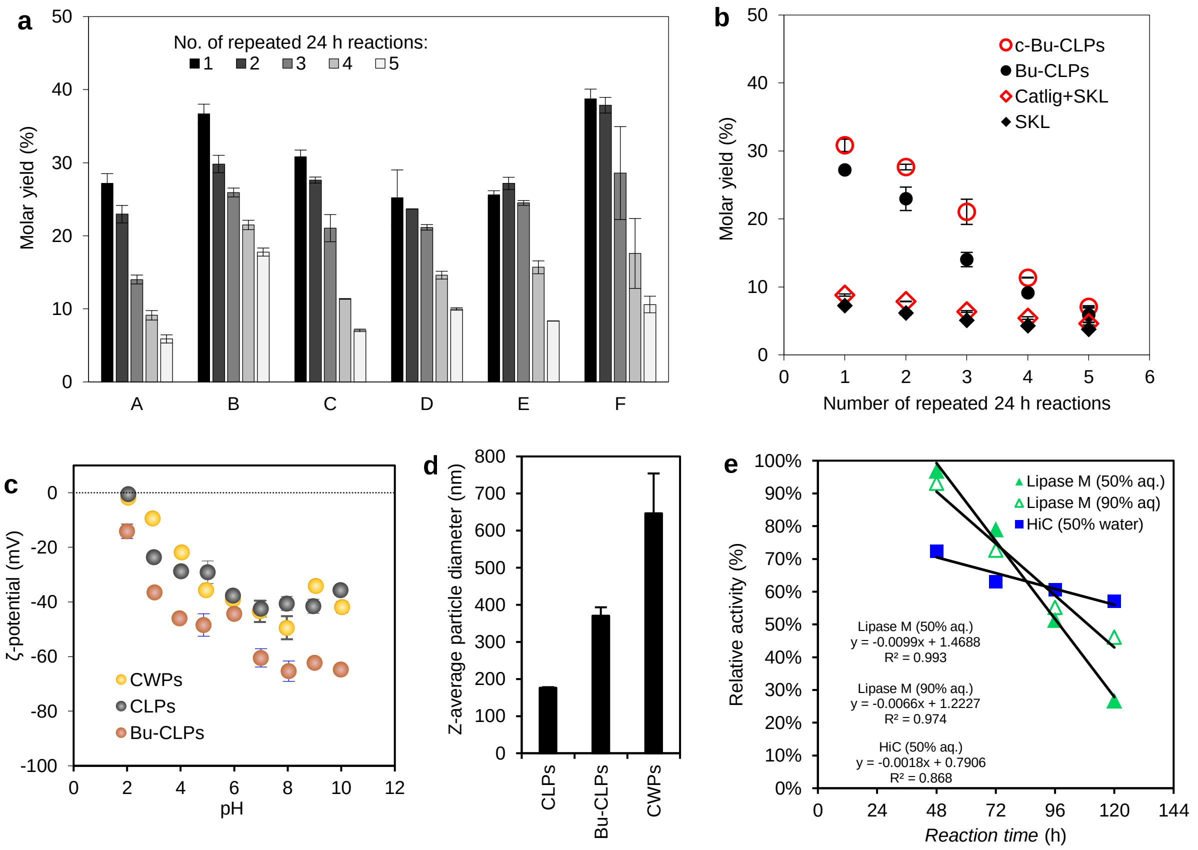

Supplementary Figure 10 | Comparison of different immobilized biocatalysts and properties of the colloidal particles used therein. a, Effect of various colloidal particles on esterification activity of Lipase M entrapped in calcium alginate matrix with or without adsorption on CLPs or CWPs in the presence or absence of Catlig. Five repeated butyl butyrate synthesis reactions were carried out at 40 ºC, hexane:water 1:1 v/v for the duration of 24 h. X-axis coding: (weight percentages in the alginate beads): A: Bu-CLP (3.6%) + Lipase M (5.4%) B: Bu-CLPs (8.4%) + Lipase M (8.3%) C: c-Bu-CLPs (3.5%) + Lipase M (5.4%) D: c-Bu-CLPs 8.3% + Lipase M 5.4% E: Catlig+CLPs+CWPs (0.3+2.0+1.0%) + Lipase M (5.1%) F: c-CLPs (0.3+3.4%) + Lipase M (5.4%). **b**, Adsorption of cationic lignin on SKL or Bu-CLPs and its effect on synthesis of butyl butyrate using Lipase M entrapped in calcium alginate with the aforementioned lignins. **c**, Comparison of surface charges (*ζ-potential*) of the particles. **d**, Z-average particle diameters of CLPs, Bu-CLPs, CWPs. Abbreviations: CLPs, colloidal lignin particles; c-Bu-CLPs, cationic CLPs prepared from butyrated lignin; c-CLPs, cationic colloidal lignin particles; c-CWPs, cationic Carnauba wax particles; Catlig, cationic lignin; SKL, softwood kraft lignin. **e**, Loss of synthetic activity of volume-confined Lipase M and HiC as a function of time. Relative activity was calculated in proportion to the activity observed after the first 24 h reaction. Error bars represent two standard deviations from the mean values (**a–b**, **e**: two experimental replicates, **c–d**: three measurements on a single experimental replicate).

In addition to the assessment of various surface active materials (lignin, CLPs, surfactants) entrapped with Lipase M for esterification reactions (Figure 2a–b in the main article),

immobilization of a microbial cutinase (HiC) was made using the developed spatial confinement method (c-CLPs as anchors) in comparison to the entrapment with Tween 80 in calcium alginate. The molar yields of butyl butyrate were essentially similar with the biocatalysts activated by c-CLP or Tween 80 (Supplementary Figure 11). Unlike with Lipase M, with HiC there was a drastic loss of activity after the first 24 h reaction. Reasons for this phenomenon observed with both c-CLP and Tween 80 containing systems are not known; however, synthetic activity showed only minor decrement after the first reaction step.

CALB adsorbed on acrylic resin allowed synthesis of butyl butyrate in 101% molar yield, with only a minor drop to 96% after five 24 h reactions (Supplementary Figure 11). However, it was not the aim to compare directly the synthetic performance of the two very different hydrolases immobilized in different carrier materials. The macroporous acrylic resin used in immobilization of CALB has known shortcomings,[12] and as presented in the main article (Figure 2c), the adsorption-entrapment method maintains synthetic activity better under water excess. Despite dosing the enzymes guided by their hydrolytic activities, the actual synthetic activities may differ notably between these enzymes. The molar yield of butyl butyrate from a 24 h reaction under similar test conditions as with CALB was increased to 82.4±1.7% when a fourfold amount of spatially confined HiC (adsorbed on c-CLPs prior to the entrapment in calcium alginate) was used (Supplementary Figure 11.) This magnitude of yield increase in response to increased biocatalyst dosing is higher than that detected with Lipase M (only 19% yield increase in 48 h reaction when the catalyst dosing was increased threefold). The main difference between these two hydrolases is the higher purity and specific activity (Supplementary Table 3), of HiC. This enabled using a significantly lower amount of alginate beads (20–80 mg, dry basis) in comparison to the amount with immobilized Lipase M (400–1200 mg, dry basis).

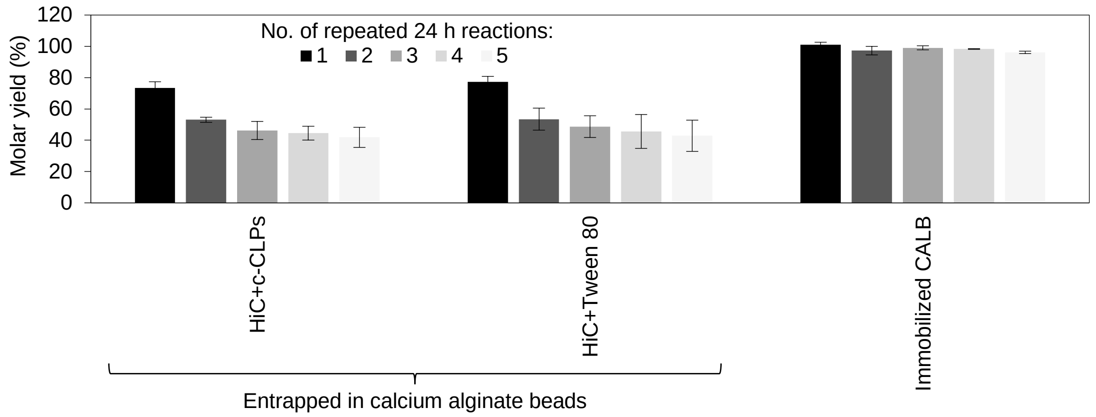

Supplementary Figure 11 | Molar yields of butyl butyrate with immobilized biocatalysts. HiC was mixed with Tween 80 or c-CLPs and subsequently entrapped into calcium alginate beads. Molar yields are shown in comparison to those from the reaction catalyzed by *Candida antarctica* lipase B (CALB) immobilized on acrylic resin (5 mg). Repeated biphasic 24 h reactions were carried out at 40 ºC. The solvent mixture comprised 2.5 ml of n-hexane and 2.5 ml of aqueous solution of 0.05 M butyric acid and 0.15 M n-butanol at pH 3. The amount of alginate beads containing immobilized HiC was 20 mg (dry basis), containing 3% (0.6 mg) of enzyme. Error bars represent two standard deviations from the mean values (two replicates).

Stability of spatially confined HiC was assessed by repeating the enzymatic butyl butyrate synthesis after 8 months (251 days) storing of the biocatalysts at 4 ºC (Supplementary Figure 12a). The reduction of synthetic activity was 29.4% relative to the molar yield obtained with the fresh biocatalyst. One reason for this high extent of activity retention was the drying and the presence of cationic lignin nanospheres that reduce water activity and risk for microbial contamination. As discussed above, drying also stabilized the alginate beads and rendered them water-resistant, with only minor leaching of protein observed at pH 2–10.5 (Supplementary Figure 12b). In contrast, the extent of protein release from the wet hydrogel beads increased from 1.5% (pH 2) to 21% (pH 10.5).

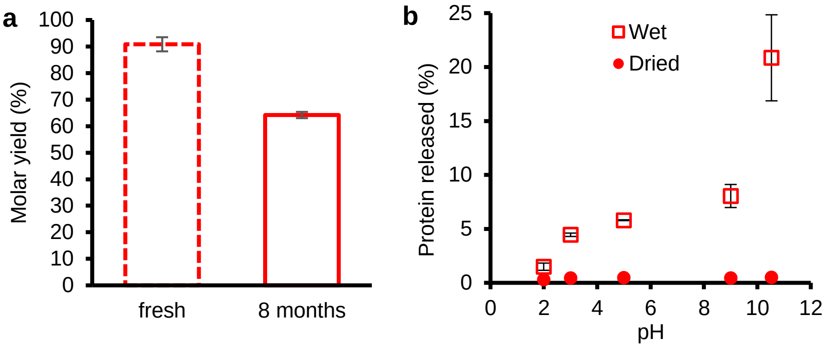

Supplementary Figure 12 | Stability of spatially confined hydrolases. a, Long-time storage stability of immobilized HiC: molar yield of butyl butyrate using freshly prepared and 8-months stored (4 °C) biocatalysts in the biphasic (hexane-water 1:1 v/v) reaction. **b,** Leaching of enzymes from the wet hydrogel beads and dried biocatalysts during 72 h magnetic stirring (120 rpm) at 21 °C in 0.2 M aqueous buffers solutions (HCl–KCl, HCl–glycine, sodium phosphate, sodium borate). Error bars represent two standard deviations from the mean values (two replicates).

Supplementary Note 6. Statistical analysis

A one-way analysis of variance (ANOVA) with Tukey's honest significant difference (HSD) all-pairwise comparison test at a significance level of $p < 0.05$ was made to analyze results presented in Figure 2a–b, d of the main article. The results summarized in Supplementary Table 5 indicate that the spatially confined immobilization system (incorporating c-CLPs as activating anchors for Lipase M) gave molar yields of butyl butyrate that were significantly different compared to other systems after 4–5 repeated 24 h reactions, while no statistically significant differences were observed in comparison to biocatalysts containing Lipase M entrapped with Tween 80 and Catlig after 1–3 repeated 24 h reactions. The biocatalyst containing Lipase M entrapped in calcium alginate alone was significantly different from the aforementioned top-performing immobilized biocatalysts.

Supplementary Table 5 | Statistical analysis of esterification results. One-way ANOVA with Tukey's honest significant difference (HSD) all-pairwise comparison test ($p<0.05$). Data for five repeated 24 h reactions with alginate beads containing Lipase M and one of the surface-active materials. Degrees of freedom (*df*) = 10. Italic letters *a–e* indicate statistically different groups.

Step no:	*SS*	*F*	*p*	Ca-alg.	DDMA	SDS	Tween 80	Catlig	CLPs	c-CLPs	Bu-CLPs	c-Bu-CLPs	SKL	Catlig +SKL
1	4373	216.2	<0.001	*c*	*c*	*d*	*a*	*a*	*b*	*a*	*b*	*b*	*cd*	*cd*
2	4371	183.8	<0.001	*e*	*ef*	*f*	*ab*	*bc*	*d*	*a*	*d*	*cd*	*e*	*e*
3	2611	61.1	<0.001	*e*	*e*	*e*	*bc*	*ab*	*c*	*a*	*cd*	*bc*	*e*	*de*
4	1000	38.5	<0.001	*ef*	*f*	*f*	*cde*	*ab*	*bcd*	*a*	*cd*	*bc*	*def*	*cdef*
5	299	87.8	<0.001	*e*	*e*	*e*	*cd*	*a*	*bc*	*a*	*bcd*	*b*	*d*	*cd*

SS: sum of squares; *F*: F-test score; *p*: probability for the validity of the null hypothesis that all results are statistically similar; Ca-alg: calcium alginate; DDMA: didodecyldimethylammonium bromide; SDS: sodium dodecyl sulfate; Catlig: cationic lignin; CLP: colloidal lignin particle: c-CLP: cationic colloidal lignin particle; Bu-CLP: CLP prepared from butyrated lignin: SKL: softwood kraft lignin.

Supplementary References

1. Chakar, F. S. & Ragauskas, A. J. Review of current and future softwood kraft lignin process chemistry. *Ind. Crops Prod.* **20,** 131–141 (2004).
2. Crestini, C., Lange, H., Sette, M. & Argyropoulos, D. S. On the structure of softwood kraft lignin. *Green Chem.* **19,** 4104–4121 (2017).
3. Sipponen, M. H., Smyth, M., Leskinen, T., Johansson, L.-S. & Österberg, M. All-lignin approach to prepare cationic colloidal lignin particles: Stabilization of durable Pickering emulsions. *Green Chem.* **19,** 5831–5840 (2017).
4. Faix, O. in *Methods in Lignin Chemistry* (eds. Lin, S. Y. & Dence, C. W.) 81–109 (Springer-Verlag Berlin Heidelberg, 1992).
5. Ämmälahti, E., Brunow, G., Bardet, M., Robert, D. & Kilpeläinen, I. Identification of side-chain structures in a poplar lignin using three-dimensional HMQC-HOHAHA NMR spectroscopy. *J. Agric. Food Chem.* **46,** 5113–5117 (1998).
6. Qu, C., Kishimoto, T., Kishino, M., Hamada, M. & Nakajima, N. Heteronuclear single-quantum coherence nuclear magnetic resonance (HSQC NMR) characterization of acetylated fir (Abies sachallnensis MAST) wood regenerated from ionic liquid. *J. Agric. Food Chem.* **59,** 5382–5389 (2011).
7. Hideko, I., Harumi, O., Hiroh, I. & Setsuzo, T. Studies on lipase from Mucor javanicus. I. Purification and properties. *Biochim. Biophys. Acta (BBA)/Lipids Lipid Metab.* **388,** 413–422 (1975).
8. Hunsen, M. *et al.* A cutinase with polyester synthesis activity. *Macromolecules* **40,** 148–150 (2007).
9. Kierstan, M. & Bucke, C. The immobilization of Microbial cell, Subcellular Organelles and Enzymes in Ca-Alginate Gels. *Biotechnol. Bioeng.* **19,** 387–397 (1977).
10. Valle-Delgado, J. J., Johansson, L. S. & Österberg, M. Bioinspired lubricating films of cellulose nanofibrils and hyaluronic acid. *Colloids Surfaces B Biointerfaces* **138,** 86–93 (2016).
11. Ng, C. H. & Yang, K. L. Lipase in biphasic alginate beads as a biocatalyst for esterification of butyric acid and butanol in aqueous media. *Enzyme Microb. Technol.* **82,** 173–179 (2016).
12. Sheldon, R. A. & van Pelt, S. Enzyme immobilisation in biocatalysis: why, what and how. *Chem. Soc. Rev.* **42,** 6223–6235 (2013).
